# Specific Nutrient Intake Via Diet and/or Supplementation in Relation to Female Stress: A Cross-Sectional Study

**DOI:** 10.1089/whr.2020.0035

**Published:** 2020-08-12

**Authors:** Delia McCabe, Jana Bednarz, Craig Lockwood, Timothy H. Barker

**Affiliations:** ^1^ The Joanna Briggs Institute, Faculty of Health and Medical Sciences, University of Adelaide, Adelaide, Australia.; ^2^ Adelaide Health Technology Assessment (AHTA), School of Public Health, The University of Adelaide, Adelaide, Australia.

**Keywords:** adaptation, dietary supplements, female, mood disorders/epidemiology, psychological, psychological/psychology, stress

## Abstract

***Background:*** Women are negatively impacted by psychological stress and despite the prolific use of dietary supplements to manage stress there is little evidence to support their use for such. This study examined the relationship between intake of specific nutrients through diet and/or dietary supplementation and level of perceived stress.

***Method:*** In this cross-sectional study of adult Australian women (*n* = 74), perceived stress was measured using the Perceived Stress Scale, dietary intake was assessed using a validated Food Frequency Questionnaire, and supplement usage was recorded using a Supplement Use Questionnaire.

***Results:*** Potentially substantive reductions in stress scores were associated with polyunsaturated fatty acid supplementation: α-linolenic acid (mean difference [MD] = −3.34, 95% confidence interval [CI] = −7.97 to 1.29), linoleic acid (MD = −4.08, 95% CI = −8.97 to 0.82), γ-linolenic acid (MD = −2.23, 95% CI = −7.20 to 2.74), and eicosapentaenoic acid (EPA)/docosahexaenoic acid (DHA) (MD = −4.05, 95% CI = −8.07 to −0.03). There were negative correlations between intake of vitamin B6 and vitamin C and stress (*ρ* = −0.50 and −0.35, respectively). Compared with nonsupplementers, stress scores were on average 0.92 units lower among those supplementing with magnesium and vitamin B6 concurrently (95% CI = −3.88 to 2.03). An increase in vitamin B6 through food was related to lower stress scores. For most nutrients, intake from food was positively associated with supplementation status.

***Conclusion:*** There is some evidence to suggest potentially meaningful associations between intake of particular nutrients and stress, although CIs were wide and there were no statistically significant relationships observed. Further research is warranted to investigate any potential benefits more precisely using randomized controlled trials or large-scale observational studies.

## Background/Introduction

Psychological stress negatively impacts a significant number of women globally, with population level studies consistently reporting higher stress levels among women relative to men.^1–3^ Psychological stress is defined as “a particular relationship between the person and the environment [appraised] as taxing or exceeding their resources and endangering wellbeing” (p. 19).^4^ Excessive or prolonged (chronic) stress impairs emotional, physical, cognitive, and social functioning and is a risk factor for the development of affective disorders.^5^ Examination of factors that are potentially protective against chronic stress, particularly among women where the burden of affective disorders is 50% higher relative to men, is warranted.^6^

Several factors may contribute to women's heightened experience of stress compared with men. These include psychosocial factors, such as persistent time constraints imposed on women by increasingly complex and competing societal roles and culture-specific stressors.^7–10^ Advances in the development of imaging technologies have also revealed gender differences in brain function and neurochemistry, including within the limbic system.^11,12^ Therefore, stress-related neurobiological mechanisms may differentially affect women and men. Such mechanisms include the development of a dysregulated stress response, whereby ongoing psychological stressors may chronically stimulate the synthesis and release specific hormones and cellular mediators aimed at mobilizing energy to address ongoing perceived threats.^13–15^ Chronic stress impacts several brain regions, including those involved in affective processing, such as the amygdala, hippocampus, and prefrontal cortex.^16^ Indeed, women are more susceptible to affective disorders than men^17^ and also more likely to become depressed after stressful life events.^18^

There is biochemical evidence for the critical importance of specific nutrients for optimal central nervous system functioning, which includes energy metabolism, affective processing, cognitive functioning, hormone and neurotransmitter synthesis, and stress response regulation.^19–23^ Central to the body's stress response is the upregulation of stress hormones, such as cortisol, synthesis of which takes precedence over that of neurotransmitters regulating mood, appetite, and sleep, such as dopamine, serotonin, and melatonin.^24–26^

Nutrient requirements are increased under chronic stress, such that demand may exceed supply, and capacity for synthesis of neurotransmitters for affective regulation reduced.^24,27,28^ Furthermore, the body's return to homeostasis after a stressful period may be delayed or compromised by nutrient deficiencies.^15^ It is suggested that when under stress women's food preferences shift toward more calorie-dense and nutrient-deficient foods, increasing their susceptibility to nutritional deficiencies.^29,30^ This may impact neurotransmitter and hormone synthesis that require cofactors, including vitamins B and C, magnesium, and zinc.^20,21,31^ In addition, polyunsaturated fatty acids (PUFAs) are critical for optimal neuronal functioning and energy metabolism.^32,33^

A cross-sectional study by Begdache et al.^34^ reported differences between men and women in the relationship between nutrient deficiency and mental wellbeing. A recent systematic review and meta-analysis found women reported significantly greater benefits from dietary interventions for symptoms of depression and anxiety compared with men^35^ and a year-long multivitamin and mineral (MVM) intervention was linked with mood improvement among women only.^36^ These results suggest that women may uniquely benefit from improved nutrient intake in the presence of stress, which may affect both nutritional choices and risk of nutrient deficiencies.

Although specific dietary patterns (DPs) have been linked with improvements in mental health,^37–39^ the impact of nutrient intake itself on perceived stress among women is presently under-examined. To date, just three studies examining the association between such nutrients and stress have included women in their study populations. In mixed-gender samples, Schlebusch et al.^40^ found significant reductions in stress levels associated with 30 days of supplementation with a B-vitamin complex, and Stough et al.^41^ reported a significant decrease in personal strain after supplementation with a high dose B vitamin complex for 12 weeks. In an all-female sample, Haskell et al.^42^ reported 9 weeks of MVM supplementation was associated with a reduction in self-reported stress levels.

Despite limited evidence, consumer expenditure on supplements claiming to be stress lowering, including B vitamins, vitamin C, and magnesium, is expected to reach USD16.7 billion by 2025.^43^ In addition, use of vitamin and mineral supplements is higher among people with a history of anxiety and/or depression, among other health challenges.^44^ Kessler et al.^45^ reported that complementary or alternative therapies, including dietary supplement (DS) use, were estimated to be used by more than half of all the individuals diagnosed with anxiety or mood disorders in a population level study. Several large-scale surveys have found dietary supplementation to be more prevalent among women compared with men.^46–48^ Given the higher levels of stress reported by women, they may also be more likely to use DSs for stress reduction compared with men.

Nutrient sufficiency during periods of stress may provide a neurobiological defense against the development of affective disorders with recent evidence suggesting specific DPs may have a preventative effect on depression.^38,49^ Such research examined the role of nutrition in mental health by measuring DPs, where effects may be attributed to interactions between nutrients that occur with the consumption of whole foods. Nonetheless, the ubiquity and popularity of nutrient-specific products claiming stress reduction suggest supplementation with isolated nutrients is perceived effective by consumers. The lack of evidence to support the clinical use of supplements for the amelioration of stress led the researchers to examine relationships between specific nutrients consumed through diet and/or DSs and perceived stress levels in women.

## Methods

### Ethics

Full ethical disclosure can be found in the Ethical Standards Disclosure section.

### Study population

This was a cross-sectional study, conducted online. A total of 87 women (21–70 years living in Queensland, Australia) were recruited, of which 74 completed the entire study (85% completion rate). Recruitment occurred through convenience sampling, through an online radio blog post, a city council newsletter, peers, and friends. Female residents of the Gold Coast were eligible to participate if they were 18 years or older at the time of recruitment, fluent in English, and could provide informed consent. Those who did not satisfy any one or more of these criteria were ineligible. Participants were required to complete three online surveys and could communicate with the researcher through a dedicated Facebook page or through email.

### Study design

Participants were emailed URL links to three survey questionnaires through SurveyMonkey on the same day they supplied their email address^50^ (SurveyMonkey, Inc., San Mateo, CA). Data were collected over a 6-week period from August 12, 2016 to September 23, 2016.

### Perceived stress assessment

Perceived stress levels were assessed using the self-report Perceived Stress Scale (PSS-10) questionnaire, which is validated for use in nonclinical populations.^51^ PSS-10 scores were automatically calculated within the SurveyMonkey software.

### Dietary assessment

An updated version of the Dietary Questionnaire for Epidemiological Studies **(**DQES version 3.2) was used to estimate nutrient intake over the past 12 months by collecting food frequency information.^52^ The DQES, referred to as a Food Frequency Questionnaire (FFQ), was validated for assessing habitual dietary intake in the Australian population.^53^ Participant's usual intake of up to 152 foods and six alcoholic beverages were measured using 37 questions. Most food items used a 10-point scale and responses were guided by food portion images and beverage consumption guides. All frequency responses were converted to estimated daily nutrient equivalents using nutrient composition databases.^54–56^

### Supplement use assessment

A self-administered Supplement Use Questionnaire (SUQ) was developed for this study by the first author (Supplementary Fig. S1). Existing questionnaires (Dietary Supplement Questionnaire [DSQ],^57^ Supplement Frequency Questionnaire [SFQ],^58^ and Vitamins and Lifestyle [VITAL])^59^ were deemed inappropriate as they referred to specific supplements only and/or were insufficiently detailed with respect to usage, motivations for use, perceived effectiveness, and purchasing behavior. To construct a comprehensive list of the composition, form, dose, and usage instructions for readily available supplements, the first author visited a pharmacy and health store and examined the labels of ∼100 DSs (including both MVMs and single-nutrient DSs). Questions were also developed to ascertain perceptions and behaviors associated with supplementation, including motivation for use and perception of effectiveness. Most questions were multiple choice. A screening question was included to establish respondent eligibility and instructions for completion were developed.

Pretesting of the questionnaire indicated face validity. A pilot test (feasibility study) was then administered using 10 purposively sampled women, including a dietician and a pharmacist, with feedback indicative of both face and content validity. Next, a specific face validity test was administered, using two groups of purposively sampled women, where one group had seen the SUQ before (*n* = 20) and the other had not (*n* = 20). There was high percentage of positive responses in both groups (95% and 90%, respectively) for questions assessing face validity.

The SUQ was adapted for use in SurveyMonkey.^50^ Information on usage and perceptions with respect to each supplement reported by each participant were obtained.

### Statistical analysis

Participant characteristics and questionnaire responses were summarized using means and standard deviations (SDs) for continuously measured variables, and frequencies and percentages for categorical items. Stress scores for supplementers and nonsupplementers were compared using the Mann–Whitney *U* test. Age-adjusted associations between supplementation status for each nutrient and PSS scores were assessed using linear regression, and Spearman's rank correlations were used to test for associations between reported nutrient intakes and PSS scores. The relationship between nutrient intakes from food and supplementation status was examined using binary logistic regression. Where multiple outcomes were assessed, Sidak's adjustment was applied to control the Type I error rate.^60^ All analyses were performed using Stata (version 15).^61^ The level of statistical significance was set to 0.05.

## Results

Table 1 describes the demographic data of survey participants including their reported supplement use. Participants used between one (*n* = 22 participants) and five supplements (*n* = 3 participants) (Supplementary Tables S1, S2, S3S5).

**Table 1.** Demographic Data, Number of Dietary Supplements Used, and Summary Statistics for Perceived Stress Scale Scores

Participant characteristics	
*N*	74
Age in years (mean ± SD)	42.7 ± 13.8
Supplement use	
Yes, *n* (%)	58 (78.4)
Total number of supplements used, *n* (%)	
1	22 (29.7)
2	16 (21.6)
3	10 (13.5)
4	2 (2.7)
5	3 (4.1)
N/A (No DS used or nutrients not of interest)	21 (28.4)
Summary statistics for PSS scores	
Mean ± SD	19.66 ± 5.90
Median	19
Min	10
Max	36

DS, dietary supplements; PSS, Perceived Stress Scale; SD, standard deviation.

PSS scores ranged from 10 to 36 (Table 1). Mean PSS scores were slightly lower among women who used DSs (M = 19.2, SD = 5.8) compared with women who did not use DSs (M = 21.4, SD = 6.3). The Mann–Whitney *U* test indicated that differences in stress scores between groups were not statistically significant (*Z* = 1.20, *p* = 0.231).

Effect of supplementation status for each nutrient on PSS scores: The age-adjusted point estimates and 95% confidence intervals (CIs) indicate that potentially substantive reductions in PSS scores were associated with the use of specific nutrients including α-linolenic acid, linoleic acid, γ-linolenic acid, and eicosapentaenoic acid (EPA)/docosahexaenoic acid (DHA) (Table 2). However, there is no consensus on what constitutes a clinically meaningful change in PSS scores. For most nutrients, average differences in PSS scores according to supplementation status were very small.

**Table 2.** Results of Linear Regression Models for Perceived Stress Scale Scores According to Supplementation Status for Nutrients of Interest

Nutrient	Coefficient (adjusted for age)	Standard error	95% CI		Unadjusted p-value	Sidak-adjusted p-value
			Lower	Upper		
α-linolenic acid^*^	−3.34	2.32	−7.97	1.29	0.154	0.886
Linoleic acid	−4.08	2.45	−8.97	0.82	0.101	0.775
γ-linolenic acid	−2.23	2.49	−7.20	2.74	0.375	0.996
EPA/DHA^a^	−4.05	2.02	−8.07	−0.03	0.048	0.522
Undefined *n*-3	−1.56	2.19	−5.92	2.80	0.478	0.997
Vitamin C	0.05	1.37	−2.68	2.77	0.973	0.998
Magnesium	−0.85	1.40	−3.64	1.94	0.545	0.997
Zinc	−0.85	1.45	−3.75	2.05	0.559	0.997
Thiamin (B1)	−1.27	1.41	−4.07	1.54	0.372	0.996
Riboflavin/Niacin (B2/B3)^b^	−1.14	1.42	−3.97	1.69	0.426	0.996
Pantothenic acid (B5)	−0.63	1.51	−3.65	2.38	0.678	0.997
Pyridoxine (B6)	−0.90	1.38	−3.65	1.85	0.517	0.997
Folic Acid	−0.40	1.46	−3.30	2.51	0.786	0.998
MTHFR^c^	−0.81	3.46	−7.70	6.08	0.816	0.998
Cyanocobalamin (B12)	0.24	1.38	−2.51	3.00	0.860	0.998

^*^ The coefficient for α-linolenic acid is −3.34, which means that the PSS scores are 3.34 units lower, on average, for those who supplement with α-linolenic acid compared with those who do not supplement with this nutrient. The 95% CI provides the range of plausible values for the “true” effect of α-linolenic acid supplementation on PSS scores that are compatible given the observed data. We therefore cannot rule out that the “true” effect of α-linolenic acid supplementation is as extreme as a 7.97 unit decrease in PSS scores and we cannot rule out that the “true” effect of such is a 1.29 unit increase in PSS scores (95% CI = −7.97 to 1.29) and the “true” effect may lie anywhere between these two values.

^a^ Model estimates for EPA and DHA use are identical, because every person who was an EPA user was also a DHA user.

^b^ Model estimates for riboflavin and niacin use are identical, because every person who was a riboflavin user was also a niacin user.

^c^ Activated folic acid supplement.

CI, confidence interval; DHA, docosahexaenoic acid; EPA, eicosapentaenoic acid; MTHFR, methylenetetrahydrofolate reductase.

Nutrient intake from DSs and PSS scores: To assess whether there is any association between nutrient dosage and stress levels among regular supplementers, correlations between nutrient intake from supplements and PSS scores were carried out for those nutrients for which there were at least 10 supplementers among the sample with daily intake data available (Table 3). Results indicated positive correlations between EPA/DHA intake and PSS scores (*ρ* = 0.79), and weak negative correlations between vitamin C intake and PSS scores (*ρ* = −0.35) and between vitamin B6 intake and PSS scores (*ρ* = −0.50). The Sidak correction was applied to account for multiple testing. The seemingly conflicting results for EPA/DHA supplementation compared with PUFA supplementation (Table 2) may be attributed by a number of factors, including uncertainty around the temporal relationship between perceived stress and supplementation.

**Table 3.** Spearman's Rank Correlations for Daily Nutrient Intake Through Dietary Supplements and Perceived Stress Scale Scores for Selected Nutrients of Interest (for *n* ≥ 10)

Nutrient	No. of observations	Rho (ρ)	Unadjustedp-value	Sidak-adjusted p-value
α-linolenic acid (mg/day)	23	0.1157	0.5991	0.9947
Linoleic acid (mg/day)	11	0.0780	0.8197	0.9947
EPA (mg/day)	10	0.7903	0.0065	0.0753
DHA (mg/day)	10	0.7903	0.0065	0.0753
Vitamin C (mg/day)	33	−0.3453	0.0491	0.3956
Magnesium (mg/day)	29	−0.1035	0.5930	0.9947
Zinc (mg/day)	22	−0.2444	0.2730	0.9220
Thiamin (B1) (mg/day)	25	−0.0296	0.8885	0.9947
Riboflavin (B2) (mg/day)	23	−0.3533	0.0982	0.6055
Niacin (B3) (mg/day)	24	−0.0931	0.6654	0.9947
Pantothenic acid (B5) (mg/day)	20	−0.1504	0.5268	0.9947
Pyridoxine (B6) (mg/day)	29	−0.4971	0.0060	0.0750
Cyanocobalamin (B12) (mg/day)	27	−0.1249	0.5347	0.9947

Supplementation with specific nutrient combinations and PSS scores: The effect of concurrent magnesium and vitamin B6 supplementation on PSS scores was examined using linear regression. PSS scores were estimated to be 0.92 units lower on average compared with participants who did not supplement with either nutrient, but the difference was not statistically significant (95% CI = −3.88 to 2.03; *p* = 0.536).

Correlation between nutrient intake from food and PSS scores: There was limited evidence for any monotonic relationships between nutrient intake through food and PSS scores (Table 4).

**Table 4.** Spearman's Rank Correlations for Daily Intake of Selected Nutrients of Interest Via Food and Perceived Stress Scale Scores (*N* = 74)

Nutrient	Rho (ρ)	Sidak adjusted p-value
Total *n*-3, mg/day	0.0291	1.000
Total *n*-6, mg/day	−0.0963	1.000
Long chain *n*-3	0.0765	1.000
Vitamin C, mg/day	−0.1891	0.925
Magnesium, mg/day	−0.0749	1.000
Zinc, mg/day	0.1390	0.997
Thiamin (B1) mg/day	−0.0206	1.000
Riboflavin (B2) mg/day	−0.0542	1.000
Niacin (B3) preformed, mg/day	0.0222	1.000
Niacin (Vitamin B3 + tryptophan derived), mg/day	0.0508	1.000
Pantothenic acid (B5), mg/day	−0.0889	1.000
Pyridoxine (B6), mg/day	−0.1335	0.998
Biotin (B7), mg/day	−0.2020	0.889
Folic acid, mg/day	0.0519	1.000
Folate, μg/day	−0.0234	1.000
Total folates, μg/day	0.0126	1.000
Dietary folate, μg/day^a^	0.0085	1.000
Cobalamin (B12), mg/day	0.1989	0.894

^a^ Combined with estimate of higher bioavailability of folic acid.

Nutrient intake from food and PSS scores: A 10 mg increase in vitamin B6 from food was associated with an average decrease of ∼3.5 units in PSS score, although inspection of the 95% CI indicates a high level of uncertainty for this effect (95% CI = *−*28.43 to 21.47). The point estimates and associated CIs for the other nutrients of interest reveal relatively small effects of nutrient intake on PSS (Table 5).

**Table 5.** Results of Linear Regression Models for Perceived Stress Scale Scores for Intakes of Selected Nutrients of Interest from Food

Nutrient	Coefficient (adjusted for age)	Standard error	95% CI		Unadjusted p-value	Sidak-adjusted p-value
			Lower	Upper		
α-linolenic acid (per 1000 mg)	0.298	1.187	−2.070	2.666	0.803	1.000
Linoleic acid (per 1000 mg)	−0.072	0.138	−0.346	0.202	0.602	1.000
EPA (per 100 mg)	0.352	0.571	−0.787	1.490	0.540	1.000
DHA (per 100 mg)	0.172	0.291	−0.409	0.753	0.558	1.000
Total *n*-3 (per 500 mg)	0.243	0.482	−0.719	1.205	0.616	1.000
Total *n*-6 (per 500 mg)	−0.034	0.069	−0.172	0.103	0.619	1.000
Vitamin C (per 25 mg)	−0.273	0.230	−0.732	0.187	0.241	0.988
Magnesium (per 10 mg)	−0.022	0.039	−0.010	0.056	0.582	1.000
Zinc (per 10 mg)	2.004	2.161	−2.304	6.313	0.357	0.999
Thiamin (B1) (per 1 mg)	0.051	0.830	−1.602	1.705	0.951	1.000
Riboflavin (B2) (per 1 mg)	0.202	0.762	−1.319	1.722	0.792	1.000
Niacin (B3) (per 10 mg)	0.500	0.844	−1.183	2.182	0.556	1.000
Pantothenic acid (B5) (per 1 mg)	0.355	0.562	−0.765	1.478	0.529	1.000
Pyridoxine (B6) (per 10 mg)	−3.481	12.513	−28.432	21.470	0.782	1.000
Biotin (B7) (per 1 mg)	−0.036	0.040	−0.115	0.044	0.378	0.999
Total folates (per 100 μg)	0.069	0.347	−0.623	0.761	0.842	1.000
Cyanocobalamin (B12) (per 1 μg)	0.646	0.390	−0.132	1.423	0.102	0.839

Nutrient intake from food and DS status: With the exception of vitamin C and vitamin B6, on average, as intake of a nutrient from food increased so too did the odds of supplementing with that nutrient. However, after adjustment for multiple comparisons, there was little evidence against a null hypothesis of no effect of intake of food on supplementation status for any nutrient. The age-adjusted odds ratios describe the change in the odds of supplementing with a particular nutrient associated with an increase in intake of that nutrient from food (Table 6).

**Table 6.** Results of Logistic Regression Models for Supplementation Status for Selected Nutrients According to Recorded Intake from Food

Nutrient (obtained through food)	Odds Ratio (age-adjusted)	Standard error	95% CI		Unadjusted p-value	Sidak-adjusted p-value
			Lower	Upper		
*α*-linolenic acid (per 1000 mg)	1.310	0.866	0.359	4.789	0.683	0.943
Linoleic acid (per 1000 mg)	1.105	0.090	0.943	1.296	0.217	0.851
EPA (per 100 mg)	2.196	0.855	0.996	4.839	0.051	0.466
DHA (per 100 mg)	1.368	0.231	0.983	1.904	0.063	0.511
Total *n*-3 (per 500 mg)	1.130	0.275	0.701	1.821	0.616	0.943
Vitamin C (per 25 mg)	0.932	0.077	0.793	1.095	0.391	0.918
Magnesium (per 10 mg)	1.030	0.015	1.001	1.060	0.043	0.435
Zinc (per 1 mg)	1.253	0.116	1.046	1.502	0.014	0.191
Thiamin (B1) (per 1 mg)	1.319	0.384	0.746	2.335	0.341	0.918
Riboflavin (B2) (per 1 mg)	1.553	0.432	0.900	2.680	0.114	0.664
Niacin (B3) (per 10 mg)	1.699	0.532	0.920	3.139	0.090	0.611
Pantothenic acid (B5) (per 1 mg)	1.094	0.230	0.724	1.653	0.671	0.943
Pyridoxine (B6) (per 1 mg)	0.728	0.341	0.291	1.824	0.499	0.937
Total folates (per 100 μg)	1.173	0.150	0.913	1.506	0.212	0.851
Cyanocobalamin (B12) (per 1 μg)^a^	1.461	0.230	1.073	1.989	0.016	0.202

^a^ For example, for every 1 μg increase in vitamin B12 intake from food, the odds of supplementing with B12 increased.

### Supplement use characteristics

The most frequently reported supplements were PUFAs that accounted for ∼23% of the 107 DSs reported, followed by MVMs, which comprised 16% of reported supplements. Vitamin C, magnesium, and vitamin B12 accounted for 13.1%, 9.4%, and 6.5% of supplements, respectively.

Most supplements (65/107 = 61%) were reportedly consumed once daily. More than 60% of the supplements were reported to have been taken for at least 6 months duration and 38.7% had been taken for >1 year.

Fifty-five percent of reported supplements were perceived as being useful by their user, whereas the efficacy of 39% of supplements was perceived as “uncertain.” The remaining 5% of supplements were perceived as not being useful. The most commonly reported motivations for DS use were to “increase energy” (applicable to 35% of supplements), “prevent disease” (applicable to 29% of supplements), and “reduce stress” (applicable to 27% of supplements) (Table 7).

**Table 7.** Summary of Associated Dietary Supplement Use Variables

Characteristic	Frequency	%
Is the supplement helping achieve goals? (*n*, %)		
Yes	59	55.1
No	6	5.6
Unsure	42	39.3
Reason for taking supplement, (*n*, %)		
Increase energy	37	34.6
Lose weight	9	8.4
Prevent disease	31	29.0
Reduce stress	29	27.1
Improve memory/concentration	20	18.7
Other	61	57.0
What prompted use of the supplement? (*n*, %)		
Doctor	31	29.0
Naturopath	15	14.0
Magazine	13	12.2
Newspaper	1	0.9
Ad	0	0.0
Other	59	55.1
Where is the supplement purchased? (*n*, %)		
Health store	38	35.5
Supermarket	25	23.4
Naturopath	5	4.7
Online	8	7.5
Doctor	5	4.7
Other	37	34.6

## Discussion

### Summary of main findings in relation to previous research

In this study, mean PSS scores were generally lower among those who used DSs compared with those who did not and average PSS scores differed according to the number of DSs reportedly used. Among supplementers, a higher intake of vitamin B6 was associated with lower PSS scores, and similarly, increased intake of vitamin B6 from food was associated with a reduction in stress score. It was found that supplementing with magnesium and vitamin B6 in combination was linked with an approximate 1 unit reduction in PSS scores. Although these associations were not statistically significant after adjustment, the findings were broadly consistent with previous studies that have shown a B complex multivitamin reduced stress in women when administered continuously over a 30-day^40^ or 90-day period^41^ and existing literature suggested that magnesium may exert stress-lowering effects.^62–64^ Many of the supplements accessed by participants contained magnesium, with more than half of all supplementers (31/58) obtaining magnesium through DSs. This may partly account for the lower stress levels observed among supplementers relative to nonsupplementers.

The effects of other B vitamins were inconclusive in this study. This may be owing to confounding by dose and duration of supplementation, which could not be completely controlled for, and low study power to detect small effects. Furthermore, this study investigated the effects of each individual B vitamin rather than a B vitamin complex, as the compositions of commercially available B vitamin complexes were highly variable. Effectiveness of supplementation may be contingent upon duration of supplementation, and any benefits may take time to have noticeable effects. Studies have reported reductions in self-reported stress associated with continuous MVM supplementation among women over time periods ranging from 9 weeks^42^ to 1 year.^36^ Reports of stress reduction after nutrient intervention may be because of the provision of a source of nutrients in short supply owing to appetite changes and increased metabolic demands that women experience when feeling stressed.^29,30^

Apart from being the most supplemented nutrient, the results indicated large potential benefits of some of the PUFA supplements with respect to stress levels. On average, stress levels were lower among those who supplemented with PUFAs compared with those who did not, with mean differences in PSS scores in the range of ∼2–4 units across all four types of such supplements. However, for the subset of supplementers who reportedly used EPA/DHA, there was a positive association between EPA/DHA dosage and PSS scores. This may indicate that while stress levels tend to be lower overall among those who supplement with PUFAs relative to those who do not, usage among the EPA/DHA supplementers may increase or decrease according to their current level of perceived stress. However, these correlations are based on only a small subset of the sample (*n* = 10) and were not statistically significant.

Although the clinical importance of differences in PSS scores of these magnitudes are yet to be empirically determined, they may represent meaningful changes in perceived stress. This is consistent with existing research indicating PUFAs may have stress-lowering effects in both genders.^65^ Because of the cross-sectional study design, a causative relationship between these variables could not be established.

With the exception of vitamins C and B6, the observed odds of supplementing with a particular nutrient increased as intake of that nutrient through food increased, although these effects were not statistically significant. This may suggest that supplementers were generally consuming higher amounts of the examined nutrients in their regular diets compared with nonsupplementers, implying that the former group is at lower risk of micronutrient deficiencies compared with the latter group. (Assessment of the nutritional adequacy of participants based on their reported food frequency intakes was beyond the scope of this study.) This result is broadly compatible with the 2013 National Health and Nutrition Examination Survey (NHANES), which found that DS use was greater among healthier respondents compared with less healthy respondents.^57^

Most DSs were reportedly consumed daily and used for at least 6 months. However, the efficacy of 39% of the supplements consumed was perceived as “unsure” and 6% were perceived as not meeting the goals of the participant. There is no research that has examined why DSs would be consumed if their efficacy were in question.

### Agreement with existing literature

Findings from this study can be compared with similar cross-sectional studies investigating dietary influences on mental health, although it should be noted that most of these studies examined food groups and DPs rather than individual nutrient intakes. Jacka et al.^37^ investigated the associations between diet (“Western” vs. “traditional”) and depression and anxiety in a sample of Australian women. Less healthy, “Western” foods were associated with increased psychological symptoms compared with more traditional diets comprising minimally processed, whole foods, including grains, fish, and meat, which are assumed to be denser in nutrients, such as those examined in this study. Jacka et al.^39^ also investigated the relationship between habitual diet and mental health in elderly men and women. A healthy DP was found to be associated with reduced anxiety, but only in women. No association was found between magnesium intake through food and depression and anxiety in further studies by Jacka et al.,^66^ and although there was some evidence for an effect of magnesium on stress in this study, this effect was relatively small in magnitude.

Previous research has indicated effects of nutrient intake on mental wellbeing may be moderated by gender.^35^ According to Begdache et al.^34^ the impact of nutrient density on mood regulation is more pronounced in women compared with men, and women may require more nutrients to support emotional wellbeing. The authors proposed that this finding may be attributable to differential effects of nutritional deficiencies on particular brain regions, such as the limbic system, between genders.^34^

### Strengths and limitations

The cross-sectional nature of this study precludes making inferences about causative relationships between variables. The majority of the sample were supplement users, which may not reflect the true proportion of users in the general population. In addition, the small sample size limited the precision of results and power, such that only very large effects would have been detected at the 5% level. Inspection of the estimated effect sizes and CIs for the specific nutrients revealed that further research involving larger samples is warranted to investigate these relationships.^67^

Although the PSS is a validated tool, the population used for validation (college students) may not be representative of the population of interest in this project.^51^ Neither is the PSS a diagnostic or screening tool, so no normative data exist and this instrument does not discriminate between acute and chronic stress.

Nutrient intake was assessed using the DQES, a high-quality, comprehensive, and well-validated questionnaire.^52^ However, over- and underreporting are inherent in all FFQs.^68^ Multiple weeks of recorded dietary intake may have provided more accurate estimates of nutrient intake.

The SUQ was not formally tested for intra- or interrater reliability. It is likely that varying degrees of recall bias were associated with each instrument.^69^

Most DSs contained more than one nutrient, which complicated the analysis of the effects of dose–response and duration of specific-nutrient consumption on stress scores. Given the small sample size, the inclusion of adjustment factors to control for confounding in models was limited. Effects exerted by single nutrients may be moderated or confounded by other factors, including intakes of other nutrients. The effects of supplementation with specific nutrients may also differ depending on a person's preexisting nutritional status, such as whether a deficiency is present.

Some research suggests that the use of DSs is higher among those with a history of affective disorders.^45^ However, no data pertaining to previous or present mental health were collected in this study. The observed effect of supplementation on PSS score may therefore be confounded by mental health status, particularly in the presence of affective disorders. Specifically, a higher prevalence of mood disorders among supplementers versus nonsupplementers may be masking potential benefits of supplementation with respect to perceived stress. The PSS scores among participants in this study may therefore not accurately reflect the relationship between these scores and supplementation, as there may be some benefit from supplementation among those with affective disorders. An inability to compare PSS scores with mental health status is therefore a limitation.

Despite these limitations, including that this study did not explore DPs or chronic stress among women, it is the first study to examine the relationship between perceived stress and the intake of individual nutrients through diet and/or DSs among women. Furthermore, it supports a developing theory that stressed women may be unlikely to voluntarily participate in health research, which suggests innovative strategies are required to gather data on this demographic.

Where possible, strategies were implemented to ensure high-quality and efficient data collection. In this study we achieved a high completion rate (85%) by using a range of recruitment and retention strategies to avoid attrition that may be relevant for other survey-based research. These strategies included engaging with local businesses and community groups who were willing to broadcast research invitations. Social media is increasingly being used to recruit participants for health research purposes and support communication toward survey completion.^70^ For this study, communication between participant and researcher was facilitated through a dedicated Facebook page. Furthermore, the development and use of the SUQ enabled information about supplement use to be readily collected without the need for structured interviews. The success of these strategies may help inform future health research.

## Conclusion

This study sought to examine the effect of specific nutrients on stress in adult women. In general, evidence for an association between any single nutrient consumed through diet and/or DSs and perceived stress was inconclusive. However, substantive reductions in stress scores associated with supplementation with PUFAs, such as α-linolenic acid, linoleic acid, γ-linolenic acid, and DHA/EPA, could not be ruled out. There was some evidence for a beneficial effect of vitamin B6 and vitamin C on stress, whereas little evidence was found to suggest supplementation with magnesium had any meaningful effect on perceived stress. Effects may have been confounded by factors including preexisting nutritional deficiency or adequacy, the presence of affective disorders, and overall diet composition.

The effect of nutritional intervention on stress is under-examined, despite chronic stress being an acknowledged antecedent of affective disorders.^5^ As similar nutrient and neurobiological mechanisms underpin mental health, further research is warranted to determine whether the effect of specific nutrient intake and/or DPs on stress is similar to their effects on depression.^38,49,71^

### Implications for future research

Prospective cohort studies or randomized controlled trials, including both genders, will allow temporal and gender effects to be examined. Results from such studies may provide evidence to support either dietary changes and/or dietary supplementation to ameliorate the negative effects of stress. Dietary supplementation is a potential intervention for stress that is relatively safe, easy to administer, and generally well tolerated. However, until more evidence is available, women in this community who use DSs to ameliorate feelings of stress may experience benefits from including stress management strategies with a more established evidence base.

## Supplementary Material

Supplemental data

Supplemental data

Supplemental data

## Abbreviations Used

CI: confidence interval
DHA: Docosahexaenoic acid
DP: dietary pattern
DQES: Dietary Questionnaire for Epidemiological Studies
DS: Dietary Supplement
DSQ: Dietary Supplement Questionnaire
EPA: Eicosapentaenoic acid
FFQ: Food Frequency Questionnaire
MD: Mean Difference
MTHFR: methylenetetrahydrofolate reductase
MVM: multivitamin and mineral
NHANES: National Health and Nutrition Examination Survey
PSS: Perceived Stress Scale
PUFA: polyunsaturated fatty acid
SD: standard deviation
SFQ: Supplement Frequency Questionnaire
SUQ: Supplement Use Questionnaire
VITAL: Vitamins and Lifestyle
