# Supplementary material for: Specific Nutrient Intake Via Diet and/or Supplementation in Relation to Female Stress: A Cross-Sectional Study
Source: Womens Health Rep (New Rochelle). 2020 Aug 12;1(1):241–51. doi: 10.1089/whr.2020.0035 (PMC7784802; doi:10.1089/whr.2020.0035)
Supplement: Supplemental data [file Supp_FigS1.pdf]

Supplementary Data

Supplement Use Questionnaire (SUQ)

Thank you for your time. This survey is about your supplementary use of dietary nutrients. A dietary supplement is a **nutritional compound that contains a nutrient/s aimed at adding extra nutritional value** to the diet, in the form of one or any combination of the following substances: a vitamin, a mineral, a herb or other botanical, an amino acid, a concentrate or an extract. This research project is specifically interested in essential fatty acids (EFAs, for example, flax or fish oil), B vitamins, vitamin C, magnesium and zinc.

Dietary supplements come in a wide range of forms, such as tablets, capsules, chewables, lozenges, gelcaps, powders, liquids, granules or others (for example, 'pearls') and injections, such as vitamin B or B12 injections. They do not include prescription medicine prescribed by your doctor or over the counter pain medication.

Please read each question carefully and answer what is true for you now in your life.

Section 1 instructions: Please read the question and tick the box that holds your answer.

Q1: Do you take any supplements or have you had any vitamin injections in the last 2 years?' ☐ Yes ☐ No

If you answered 'NO' to question 1, you have completed the questionnaire and you can ignore the rest of the questions.

Section 2 instructions: Please complete each of the sub-sections below, by writing your answers in the boxes or circling your response. Please provide details if you choose the 'other' option. This section is about the brand, type and form of the supplement/s you use, along with the regularity, quantity, and longevity of use. It also questions the reason you take the supplement/s and whether you think it/they are helping you. Each column focuses on one supplement. Please complete one column for every supplement taken.

	SUPPLEMENT 1	SUPPLEMENT 2
Supplement brand/manufacturer name - *e.g. Blackmores*<br>*Or if it is an injection, write 'injection'*		
Type of supplement - *e.g. Magnesium*<br>*Or if it is an injection, write 'B/B12 vitamin'*		
What form does the supplement come in?	Tablets Capsules Powder<br>Liquid Granules Chewables<br>Lozenges Pearls Injection<br>Other: ____	Tablets Capsules Powder<br>Liquid Granules Chewables<br>Lozenges Pearls Injection<br>Other: ____
How often do you take this supplement?	Every day 4-5 times per week 2-3 times per week<br>Once per week Once per month<br>Other: ____	Every day 4-5 times per week 2-3 times per week<br>Once per week Once per month<br>Other: ____
If you take this supplement every day, how often do you take it every day?	1 x per day 2 x per day 3 x per day<br>Other: ____	1 x per day 2 x per day 3 x per day<br>Other: ____
How many/how much of this supplement do you take EACH time *e.g. 1 tablet/capsule OR 1 tsp OR 1 Tbsp OR 1 injection*		
For how long have you taken this supplement?	Less than 1 month 1-3 months 3-6 months<br>6-12 months More than 1 year	Less than 1 month 1-3 months 3-6 months<br>6-12 months More than 1 year
Why do you take this supplement?	Increase energy levels Lose weight Prevent disease Reduce stress<br>Improve memory & concentration Other: ____	Increase energy levels Lose weight Prevent disease Reduce stress<br>Improve memory & concentration Other: ____
Is this supplement helping you achieve this goal/these goals?	Yes No Unsure	Yes No Unsure
What prompted you to start taking this supplement?	Doctor Naturopath Magazine article<br>Newspaper article Advertisement<br>Other: ____	Doctor Naturopath Magazine article<br>Newspaper article Magazine advertisement<br>Other: ____
Where do you purchase this supplement?	Health Store Supermarket Naturopath<br>Online Doctor Other: ____	Health Store Supermarket Naturopath<br>Online Doctor Other: ____

SUPPLEMENTARY FIG. S1. Supplement Use Questionnaire.
