# Supplementary material for: Specific Nutrient Intake Via Diet and/or Supplementation in Relation to Female Stress: A Cross-Sectional Study
Source: Womens Health Rep (New Rochelle). 2020 Aug 12;1(1):241–51. doi: 10.1089/whr.2020.0035 (PMC7784802; doi:10.1089/whr.2020.0035)
Supplement: Supplemental data [file Supp_TableS1-S2-S3.pdf]

Supplementary Table S1. Summary of the Distribution of Dietary Supplement Combinations Accessed by Participants Who Reported Using One Dietary Supplement ($n = 22$)

DS type	Frequency	%
Cyanocobalamin (B12)	3	13.6
Pyridoxine (B6) + magnesium + zinc	1	4.5
B vitamins	2	9.1
B vitamins + magnesium	1	4.5
PUFAs	6	27.3
Magnesium	1	4.5
Multivitamin	4	18.2
Vitamin C	4	18.2

DS, dietary supplement; PUFAs, polyunsaturated fatty acids.

Supplementary Table S2. Summary of the Distribution of Dietary Supplement Combinations Accessed by Participants Who Reported Using Two Dietary Supplements ($n = 16$)

DS type 1	DS type 2	Frequency	%
Multi-vitamin	Vitamin C	2	12.5
PUFAs	Vitamin C	2	12.5
PUFAs	B vitamins	1	6.3
PUFAs	Thiamin (B1)	1	6.3
PUFAs	Cyanocobalamin (B12)	1	6.3
PUFAs	B vitamins + magnesium	1	6.3
PUFAs	Magnesium	1	6.3
PUFAs	Cyanocobalamin (B12)	1	6.3
Multivitamin	Magnesium	1	6.3
Multivitamin	B vitamins + magnesium	1	6.3
Vitamin C	Cyanocobalamin (B12)	1	6.3
Vitamin C	Pyridoxine (B6) + magnesium	1	6.3
PUFAs	Multivitamin	1	6.3
Vitamin B + magnesium	Vitamin B + magnesium	1	6.3

Supplementary Table S3. Summary of the Distribution of Dietary Supplement Combinations Accessed by Participants Who Reported Using Three Dietary Supplements ($n = 10$)

DS type 1	DS type 2	DS type 3	Frequency	%
PUFAs	Magnesium	Magnesium	1	10.0
PUFAs	Magnesium + zinc	Vitamin C + zinc	1	10.0
PUFAs	Multivitamin	Vitamin C + zinc	1	10.0
PUFAs	PUFAs	Vitamin B6 + magnesium	1	10.0
Vitamin C	B-vitamins + vitamin C	Magnesium + vitamin C	1	10.0
Vitamin C	Multivitamin	Pyridoxine (B6) + magnesium	1	10.0
Multi-vitamin	PUFAs	Vitamin C	1	10.0
Multi-vitamin	Cyanocobalamin (B12)	Magnesium	1	10.0
B vitamins	Magnesium	Magnesium	1	10.0
B vitamins	Magnesium	B vitamins + magnesium	1	10.0
