# Supplementary material for: Specific Nutrient Intake Via Diet and/or Supplementation in Relation to Female Stress: A Cross-Sectional Study
Source: Womens Health Rep (New Rochelle). 2020 Aug 12;1(1):241–51. doi: 10.1089/whr.2020.0035 (PMC7784802; doi:10.1089/whr.2020.0035)
Supplement: Supplemental data [file Supp_TableS4-S5.pdf]

Supplementary Table S4. Summary of the Distribution of Dietary Supplement Combinations Accessed by Participants Who Reported Using Four Dietary Supplements ($n = 2$)

DS type 1	DS type 2	DS type 3	DS type 4	Frequency	%
Pyridoxine (B6) + Mag + VitC	γ-linolenic acid	VitC + zinc	VitC + zinc	1	50.0
Thiamin (B1)	Multivitamin	Cyanocobalamin (B12)	γ-linolenic acid	1	50.0

Supplementary Table S5. Summary of the Distribution of Dietary Supplement Combinations Accessed by Participants Who Reported Using Five Dietary Supplements ($n = 3$)

DS type 1	DS type 2	DS type 3	DS type 4	DS type 5	Frequency	%
PUFAs	Multivitamin	VitC	Pyridoxine (B6) + Mag	γ-linolenic acid	1	33.3
PUFAs	Multivitamin	Mag	Zinc	Folate	1	33.3
PUFAs	PUFAs	B Vits	Pyridoxine (B6) + Mag	VitC + zinc	1	33.3
